# Molecular and Genetic Profiling for Precision Medicines in Pulmonary Arterial Hypertension

**DOI:** 10.3390/cells10030638

**Published:** 2021-03-13

**Authors:** Shahood Fazal, Malik Bisserier, Lahouaria Hadri

**Affiliations:** Cardiovascular Research Institute, Icahn School of Medicine at Mount Sinai, 1470 Madison Avenue, New York, NY 10029, USA; shahood.fazal@icahn.mssm.edu (S.F.); malik.bisserier@mssm.edu (M.B.)

**Keywords:** pulmonary hypertension, precision medicine, treatment, genetics, mutation, genomic medicine, gene therapy

## Abstract

Pulmonary arterial hypertension (PAH) is a rare and chronic lung disease characterized by progressive occlusion of the small pulmonary arteries, which is associated with structural and functional alteration of the smooth muscle cells and endothelial cells within the pulmonary vasculature. Excessive vascular remodeling is, in part, responsible for high pulmonary vascular resistance and the mean pulmonary arterial pressure, increasing the transpulmonary gradient and the right ventricular “pressure overload”, which may result in right ventricular (RV) dysfunction and failure. Current technological advances in multi-omics approaches, high-throughput sequencing, and computational methods have provided valuable tools in molecular profiling and led to the identification of numerous genetic variants in PAH patients. In this review, we summarized the pathogenesis, classification, and current treatments of the PAH disease. Additionally, we outlined the latest next-generation sequencing technologies and the consequences of common genetic variants underlying PAH susceptibility and disease progression. Finally, we discuss the importance of molecular genetic testing for precision medicine in PAH and the future of genomic medicines, including gene-editing technologies and gene therapies, as emerging alternative approaches to overcome genetic disorders in PAH.

## 1. Introduction

Pulmonary arterial hypertension (PAH) is a rare but life-threatening lung disease characterized by a mean pulmonary arterial pressure (PAP) greater than 20 mmHg at rest, measured by right heart catheterization and the need for pulmonary vascular resistance (PVR) >3 wood units [1]. Vascular remodeling of the distal pulmonary arteries plays a critical role in arterial stiffening, thickening, and the increase in the pulmonary vascular resistance (PVR) [2]. Although changes in the pulmonary vasculature are the primary cause of PAH, the severity of symptoms and survival are strongly associated with RV dysfunction and RV failure, which can ultimately lead to death. While the RV shows greater compliance and adaptation to changes in volume load by increasing contractility, the RV is characterized by concentric hypertrophy at the early stage of PAH in response to an increase in afterload [3]. The elevated systolic and diastolic ventricular pressures exert a sustained mechanical and hemodynamic stress on RV by stretching the RV wall. This initially leads to an increase in the cardiac mass known initially as adaptive hypertrophy [3]. However, low oxygenation of the RV induces a series of structural and functional changes in the cardiomyocytes and the extracellular matrix, which alters the contractility of the RV leading to RV dysfunction [4]. A decrease in cardiac output in patients with severe PAH may lead to heart failure and multi-organ failure. Yet, the transition from compensated to decompensated RV hypertrophy remains poorly understood in PAH.

## 2. Classification of PAH

The World Health Organization and European Repository Journal have classified pulmonary hypertension (PH) into five groups [1,5]. Group 1 entails PAH and includes heritable pulmonary hypertension (HPAH) and idiopathic pulmonary hypertension (IPAH). HPAH is associated with various genetic mutations (i.e., Bone Morphogenetic Protein Receptor Type-2, Activin A Receptor Like Type 1, Potassium Two Pore Domain Channel Subfamily K Member 3, SMAD Family Member 9) [1]. IPAH includes cases where the underlying cause of the narrowing of the arteries is unknown. Several studies have shown an association between PAH and specific drugs as well as other underlying conditions, such as connective tissue disorders. Group 2 encompasses PH due to left heart disease and includes diseases associated with left ventricular systolic and diastolic dysfunction, valvular disease, or congenital heart defects [1]. As of today, no genetic mutation has been identified or associated with group 2 PH. Group 3 includes PH induced by lung diseases or hypoxia and includes chronic obstructive pulmonary disease (COPD), interstitial lung disease, sleep-disordered breathing, lung developmental anomalies, or alveolar hypoventilation disorders [1]. Gene polymorphism might contribute towards determining the severity of PH in hypoxemic patients with COPD [6]. Group 4 refers to PH caused by blood clots obstructing pulmonary arteries and includes chronic thromboembolic pulmonary hypertension (CTEPH) [1]. Currently, no genetic mutations have been linked to group 4 PH. Group 5 includes less-common causes and unclassified disorders. This group is widely divided into four categories: blood disorders, systemic disorders, metabolic disorders, and other disorders such as chronic kidney failure or tumors obstructing the pulmonary artery [1]. The heterogeneity of this group prevents an appropriate description of genetics or risk factors.

## 3. Physiopathology of PAH

In PAH, the entire pulmonary vascular tree undergoes alterations, and the small vessels are primarily affected [2]. Histologically, severe PAH is characterized by medial and adventitial thickening, occlusive neointima, as well as complex plexiform lesions [7]. The media thickening is, in part, responsible for increasing the PAP [7]. Pulmonary artery smooth muscle cells (PASMCs) are characterized by abnormal and excessive proliferative rates and migratory properties. Most of these changes are due to the growth factors that are overexpressed in patients with PAH, such as platelet-derived growth factor (PDGF), endothelin (ET), serotonin (5-HT), transforming growth factor-β (TGF-β), epidermal growth factor (EGF), fibroblast growth factor (FGF) [8]. The vascular muscularization reduces the diameter of the pulmonary vessels and the responsiveness to further vasodilation [7], with increased vascular wall shear stress that contributes to endothelial dysfunction. Under physiological conditions, the endothelium releases both vasoconstrictors and growth-promoting factors maintained in a balanced homeostatic state [9]. Nonetheless, endothelial dysfunction or lesions impairs the production and release of critical vasodilatory factors such as nitric oxide (NO), prostacyclin (PGI_2_), and endothelium-derived hyperpolarizing factor (EDHF) [10,11]. Currently, most medicines on the market aim to leverage these impairments by restoring the imbalance between vasoconstrictors and vasodilators.

In severe PAH, plexiform lesions are commonly found in distal muscularized pulmonary artery segments and branch points and promote disease severity through inflammatory vascular remodeling [12]. They are described as occlusive glomeruloid-like vascular lesions and morphologically complex. Increasing evidence showed that vascular injury is a determinant contributor by promoting endothelial cell survival, hyperproliferation, and inflammatory cell infiltration. Indeed, plexiform lesions represent an overgrowth of “apoptosis-resistant” endothelial cells into the lumen. The formation of plexiform lesions is also associated with apoptosis-resistant myofibroblasts, smooth muscle cells, and possibly undifferentiated mesenchymal cells [13]. They are “slit-like” channels of endothelial cells within the occluded vessel lumen surrounded by smooth muscle cells, myofibroblast, and inflammatory cells, including CD3-positive T cells and CD68-positive macrophages [13]. The remodeling process with plexiform lesions is more pronounced and intricate than precapillary vascular compartments in PAH lungs. The molecular mechanisms underlying the formation of plexiform lesions remain largely unknown. A study showed that pulmonary artery endothelial cells (PAECs) from plexiform lesions in patients with PAH had decreased expression of PHD2. This enzyme facilitates the degradation of HIF1 and HIF2 hypoxia sensors [14], known as critical contributors to pulmonary vascular remodeling in PAH.

## 4. Current Therapies in PAH

The clinical profile of PAH has tremendously changed over the past 20 years. PAH was initially described as a non-treatable disease. However, the recent advances in diagnostic tools, multi-omics technologies, and early treatment have significantly improved patients’ quality of life and overall life expectancy [15,16,17]. Current data shows that the median survival was 2.8 years between 1980 and 1991 when no specific and efficient treatments were available, while the median survival is 7–10 years in the modern treatment era [18,19].

Today, around 14 therapies are currently available and commonly used clinically. These therapies target three distinct pathways and aim to restore the balance between vasodilation and vasoconstriction [15,20]. The prostacyclin pathway and the nitric oxide-cyclic guanosine monophosphate (NO-cGMP) signaling potentiate vasodilation and attenuate the proliferation of vascular cells, including smooth muscle cells (SMCs) and endothelial cells (ECs) [21]. Thus, conventional therapies attempt to stimulate these two pathways to counteract vascular remodeling and decrease the PAP. For example, the therapies such as sildenafil, tadalafil, riociguat, epoprostenol, iloprost, and selexipag are used clinically and significantly mitigate the symptoms by reducing the vasoconstriction, vascular remodeling while favoring the vasodilation [22]. Altogether, the current treatments decrease PVR, PAP, and improve the patient’s quality of life. Similarly, as the endothelin pathway regulates vasoconstriction, ambrisentan, bosentan, and macitentan aim to limit the activation of this pathway and subsequently attenuates vasoconstriction, PVR, and PAP [23,24]. Nonetheless, these drugs have shown only short-term benefits and may cause systemic hypotension in some patients [25]. Unfortunately, the current medications fail to cure PAH despite short-term improvements.

The spectrum of therapeutic options for PAH has tremendously expanded in the past 20 years. However, all the available therapies remain indicated for palliative purposes only. New experimental approaches have expanded our possibilities of finding novel therapeutic targets and improve our understanding of PAH. Indeed, the identification of new molecular regulators and a better understanding of their role in the PAH pathogenesis have opened new avenues to treat PAH over the past decade.

## 5. Advances in Genomic Technologies

Current advances in genetic testing and computational methods have equipped researchers with innovative tools that can help identify the genetic mutations and understand their role in PAH [26]. In HPAH, genetic panel testing has been developed to help physicians with early diagnosis of PAH or identify the patients that are susceptible to developing PAH [27]. Genetic studies have provided valuable information regarding the molecular pathways that can be leveraged when developing therapies for PAH [26,27]. Several genetic approaches have been used to detect novel mutations, and further studies are currently being investigated to evaluate their relevance to PAH.

Targeted-sequencing has been crucial as the number of genes associated with PAH have increased [26,27]. Next-Generation Sequencing (NGS) has enabled the development of new gene panels that can interrogate several genes of interest simultaneously [26,27]. NGS allows the analysis of several genes for serial analysis of gene expression (SAGE) changes to detect duplication in exons [26,27]. The NGS technology is a novel and powerful tool for defining a specific genetic profile of PAH patients, analyze dysregulated molecular networks, identify altered biological mechanisms and cellular pathways. Moreover, whole-exome sequencing and whole-genome sequencing have enabled the identification of variants in the non-coding regions of the patient’s genome, which allows for the identification of novel genes as potential therapeutic targets [26,27]. These new technologies have led to the discovery of a variety of new mutations in PAH patients. Increasing evidence demonstrates that the genetic basis of PAH is of great clinical value as it may lead to the development of precision or individualized medicines and enhance our ability to cure PAH effectively.

Additionally, the implementation of complementary bioinformatics analysis has been critical in analyzing large datasets for new mutations in PAH patients. Computational software and algorithms have also provided researchers with tools necessary to detect genetic variants and single nucleotide polymorphisms. Taken together with statistical methodologies, these advances are highly valuable clinical tools and lay a foundation for further studies.

## 6. Genetic of PAH

**Figure 1:**
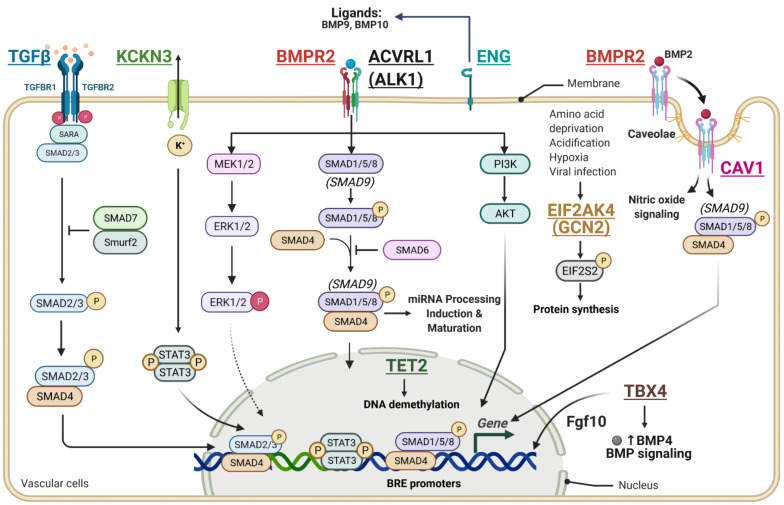
Gene variants in HPAH impairs the BMP signaling and potentiates the development of PAH. Heritable pulmonary hypertension (HPAH) is associated with various genetic mutations (i.e., BMPR2, ENG, ALK1, SMAD9, E1F2AK4, CAV-1, KCNK3, and TBX4). Genetic variants are characterized by a loss-of-function in PAH and converge within the BMP-Smad 1/5/8 signaling, increasing the proliferation, apoptosis resistance, migration in vascular cells, and the development of PAH. Dysregulation of the BMPR2 signaling impairs the MAPK and PI3K/AKT signaling. Mutations in the KCNK3 gene induce membrane depolarization and opening of voltage-gated calcium channels, which increase the free cytosolic calcium concentration in PASMCs and promote vasoconstriction as well as vascular remodeling. The loss of Cav-1 induces the disruption of caveolae, blocks the initiation of the SMAD signaling, increases endothelial nitric oxide synthase (eNOS) activity, and potentiates the development of PAH through protein kinase G (PKG) nitration. TBX4 plays a central role in the development of the respiratory tract and lung branching during embryogenesis through its interaction with fibroblast Growth Factor 10 (Fgf10). ACVRL1, activin receptor-like 1 (ALK1); Bone morphogenetic protein receptor type 2 (BMPR2); Caveolin-1 (Cav-1); Endoglin (ENG), Eukaryotic Translation Initiation Factor 2 Alpha Kinase 4 (EIF2AK4); SMAD, mothers against decapentaplegic homolog 9 (SMAD9), Signal Transducer And Activator Of Transcription 3 (STAT3); T-Box Transcription Factor 4 (TBX4).

Considering the importance of genetic mutations in PAH, it is of great clinical value to understand the consequences of these mutations in various biological processes and define their potential role in PAH. Recent studies have leveraged modern technologies, including different sequencing methods, to compile a list of genes that are mutated and their frequency in PAH. Here, we summarize the major and frequent mutations reported in PAH (Table 1) and discuss their role in the development of PAH (Figure 1).

### 6.1. Bone Morphogenetic Protein Receptor Type 2 (BMPR2)

BMPR2 is a serine and threonine receptor kinase and belongs to the superfamily of transforming growth factor β (TGF-β) [28,29,30]. Bone morphogenetic protein (BMP) ligands bind to BMPR2 and regulate paracrine signaling [28,30]. The BMP signaling regulates several cellular functions such as osteogenesis, cell growth, and cell differentiation [31]. The BMP binding to BMPR2 induces the recruitment of BMPR1 and undergoes auto-phosphorylation. BMPR1 phosphorylates the transcription regulator R-SMAD, known as receptor-regulated SMADs [30]. Studies have shown that BMPR2 is highly expressed in the pulmonary endothelium, where it forms a molecular complex with Bone Morphogenetic Protein Receptor Type 1 (BMPR1), Activin A Receptor Like Type 1 and 2(ALK1, ALK2, respectively) ALK2 in response to ligand binding. The BMP9 and BMP4 ligands both function as circulating vascular quiescence factors and protect ECs from excessive proliferation, apoptosis, and permeability [32,33].

The BMPR2 signaling showed contrasting effects in PASMCs and PAECs. In vitro studies in human PASMC have demonstrated that BMP signaling inhibits muscularization by reducing PASMC growth and increasing apoptosis while promoting proliferation and endothelial cell survival [34,35,36]. BMP4 ligand stimulation potentiates PAEC proliferation, migration, and the formation of tubular structures [37]. PAEC proliferation is mediated through the activation of SMAD1/5 and is tightly regulated by the induction of inhibitors of the DNA binding family of transcription factors. In vitro, BMPR2 overexpression or activation inhibits the proliferation of PASMC and promotes the survival of PAEC [38]. Additionally, BMPR2 suppression induced arterial damage and adverse inflammatory responses [39]. Indeed, genetic ablation of the BMPR2 gene in pulmonary endothelium is sufficient to predispose to pulmonary arterial hypertension as indicated by the increased pulmonary and RV pressures, increased number of muscularized distal arteries, and thickening of medial layers, as well as the formation of plexiform lesions [40]. Consistently, reduced BMPR2 expression, Smad signaling in lungs, and small arteries were found in animal models of PAH, such as monocrotaline- (MCT-)-induced PH in rats [41].

Genetic analysis of families with PAH identified heterozygous germline mutations in the BMPR2 gene [42]. To date, over 400 different mutations in BMPR2 have been uncovered in PAH, and mutations within the BMPR2 gene account for 75% of familial PAH and 25% of Idiopathic PAH [43,44]. Heterogeneous mutations in the BMPR2 gene disrupt the BMP and SMAD signaling and potentiate a pro-proliferative state in PASMC [45,46]. Substitution mutations within the ligand-binding (C60Y, C117Y, C118W, C123R, and C123S) or kinase domain (C347Y, C420R, and C483R) affect the subcellular localization of BMPR-2 by reducing the trafficking of BMPR2 to the cell surface in NMuMG and HeLa cells, immortalized human coronary artery smooth muscle cells (CASMCs) and PASMCs overexpressing various mutant BMPR2 constructs generated by site-directed mutagenesis [47]. Non-cysteine substitutions within the kinase domain (D485G and R491Q) reach the cell surface and fail to activate the SMAD-responsive genes due to an inability to phosphorylate BMPR2 in vitro [45]. Thus, haploinsufficiency or missense mutations seem to lead to a loss of signaling via the SMAD1/5 pathway [48].

Considering the high frequency and prevalence of BMPR2 mutations in PAH, the scientific community has been actively investigating these mutations to better understand the molecular mechanisms and their role in the setting of the disease phenotype. In vitro studies using human PAECs showed that BMPR2 mutations increase the susceptibility of PAECs to apoptosis, which may compromise the integrity of the pulmonary endothelial barrier and contributes to endothelium dysfunction [49]. Several studies demonstrated that the loss of the BMPR2 signaling exacerbates the proliferation of vascular cells, apoptosis resistance, and the transition towards a mesenchymal state in response to circulating growth factors in PAH [40,50,51]. Similarly, vascular cells carrying BMPR2 mutations are characterized by a hyperproliferative state and increased resistance to apoptosis in response to growth factors in transgenic mice carrying BMPR2 mutations [50,52,53].

### 6.2. Growth Differentiation Factor 2 (GDF2)

The GDF2 gene belongs to the TGF-β superfamily and encodes for the secreted ligand BMP9. This ligand is produced by the liver, circulates in the bloodstream, and interacts with high affinity with ALK1 in association with BMPR2 and activin receptor 2B (ACTRIIB). In physiological conditions, BMP ligands maintain vascular endothelial quiescence. Previous studies have shown that the therapeutic administration of BMP9 prevents and reverses PAH in genetic and non-genetic models of PAH. Consistently, recent studies have shown that impairment of the GDF2/BMPR2/ACRL1 signaling in PAEC plays a critical role in the pathogenesis of PAH, especially in heritable PAH.

GDF2 mutations were mostly detected in adult-onset patients with heterozygous missense variants and are associated with reduced production of GDF2 [54]. The authors performed whole-genome sequencing in 1038 PAH index cases and 6385 PAH-negative control subjects. Of the 1048 PAH cases, 908 (86.7%) were diagnosed with idiopathic PAH, 58 (5.5%) gave a family history of PAH, and 60 (5.7%) gave a history of drug exposure associated with PAH. The results revealed significant overrepresentation of rare variants in ATP13A3, AQP1, and SOX17. Importantly, Gräf and collaborators provided independent validation of a critical role for GDF2 in PAH.

Similarly, Hodgson et al. performed whole-genome sequencing using the same cohort and identified seven likely pathogenic missense variants and one frameshift variant in GDF2 in PAH patients [54]. The authors further investigated additional structural variation at the GDF2 locus and identified two patients with large deletions, not previously described, encompassing the GDF2 locus and several neighboring genes. This study identified the new variant p.Y351H in two unrelated patients, which is predicted to disrupt the hydrophobic core of BMP4 and ultimately impair the BMP9 complex. Hodgson and colleagues demonstrated that the secretion of BMP9 variants was markedly reduced in conditioned media by ELISA and western blotting in nonreducing conditions. This new pathogenic variant exhibited an excess of prodomain compared to the growth factor domain suggesting the Pro:BMP9 complex was indeed disrupted [54]. Finally, the authors also showed that patients carrying the putatively pathogenic GDF2 alleles (Pro: BMP9-M89V, -A347V,-Y351H, or -T413N) had significantly lower mean plasma BMP9 levels than age-matched controls.

Using a cohort of 120 control samples and 260 cases of heritable or idiopathic PAH, the authors found that the plasma levels of BMP9 and BMP10 are significantly higher in females than males in both control and PAH groups [54]. Levels of ligands were not associated with age. Interestingly, no differences in plasma BMP9 levels were observed either in male control or male PAH patients. However, plasma BMP9 levels were significantly higher in female patients with PAH [54].

Hodgson et al., compared BMP9 and pBMP10 tertiles with the clinical characteristics to perform correlation analysis for relationships (continuous variables) between ligand concentrations and clinical parameters. First, they found that BMP9 and pBMP10 levels were not associated with exercise capacity measured by the 6-min-walk test in patients with PAH. Analysis of clinical blood tests revealed that plasma BMP10 negatively correlated with red cell distribution width and alkaline phosphatase activity in the PAH cohort, while BMP9 levels positively correlated with platelet cell count. Both BMP9 and BMP10 also correlated negatively with C-reactive protein (CRP).

In PAH cells, previous studies identified the Interleukin-6 dependent signaling as a central mediator required for BMP9-induced phenotypic changes in PAH patient-derived cells [55]. In PAEC isolated from PAH patients, prolonged endothelial to mesenchymal transition (EndMT) signaling is accompanied by a sustained elevation in pro-inflammatory, pro-hypoxic, and pro-apoptotic signaling. Szulcek et al. targeted the BMP9-induced EndMT process to normalize autocrine IL-6 levels using an IL-6 antibody and prevent the mesenchymal transformation and maintain a functional EC phenotype. Altogether, the authors found that exacerbated IL-6-mediated inflammatory signaling underlies aberrant response to BMP9 in PAEC. Exaggerated pro-inflammatory signaling showed that BMP9-induced aberrant EndMT is dependent on PAH pulmonary ECs. This might provide a therapeutic strategy to increase GDF2 expression to restore proper BMPR2 signaling [56].

### 6.3. Endoglin (ENG)

The Endoglin (ENG) gene encodes a major glycoprotein of the vascular endothelium [57]. ENG is a component of the TGF-β receptor complex and binds to the beta1 and beta3 peptides with high affinity [57]. It is predominantly expressed in proliferative ECs and regulates several critical biological processes such as proliferation, angiogenesis, inflammation, cell differentiation, and extracellular matrix deposition [57]. In the lungs, ENG maintains a balance between ALK1 and ALK5, which regulates endothelial cell proliferation and migration. ENG and ALK1 levels are both increased in PAECs from human patients with IPAH. In PAEC, ENG overexpression promotes the induction of growth factors endothelin 1 (ET-1), platelet derived growth factor (PDGF), and Fibroblast Growth Factor 2 (FGF2) in PAECs. These growth factors have a stimulatory effect on the growth of PASMCs and are elevated in human PAH [58]. Several growth factors, including ET-1, epidermal growth factor (EGF), and vascular endothelial growth factor (VEGF), are implicated in the proliferation and migration of pulmonary vascular cells. They act as potent mitogens and chemoattractants for SMCs, and ECs. Previous studies have demonstrated their role in the resistance to apoptosis and their implication in PAH development [59].

Mutations in the ENG gene are associated with hereditary hemorrhagic telangiectasia (HHT), also known as Osler–Rendu–Weber syndrome 1. HHT is an autosomal dominant multisystemic vascular dysplasia [60,61,62,63,64], which is characterized by the presence of abnormal connections between the arteries and veins, also known as arteriovenous malformations. Genetic evidence suggests that mutations in the ENG gene in pulmonary ECs may trigger the onset of PAH by impairing the intracellular signaling [60,65]. Studies have shown that mutation within the ENG gene may predispose patients with HHT to PH by dysregulating the TGF-β signaling [59,66,67,68]. Currently, 15 variants of the ENG gene have been identified in HTT patients [65]. The exons 6 and 12 are considered hotspots for pathogenic mutations [65]. While there is no direct interaction between BMPR2 and ALK1/ENG complexes, the existence of signaling crosstalk may be extremely important as they tightly regulate the cellular homeostasis through common effectors, including the SMAD proteins 1, 5, and 8 [59]. Further investigations remain necessary to elucidate the role of these mutations on the downstream pathways and better understand their impact on the setting of PAH.

### 6.4. Activin A Receptor, Type II-Like Kinase 1 (ALK1 or ACVRL1)

Activin A receptor, type 1-like kinase 1 (ALK1 or ACVRL1), is a serine and threonine receptor kinase and acts as a type 1 receptor for the TGF-β/BMP superfamily of ligands [69]. Similarly to BMPR2 signaling, ligand binding activates the TGF-β pathways and potentiates the formation of a molecular complex involving Type 1 and Type II serine and threonine receptors at the cell surface [70]. In ECs, this process is facilitated by the formation of the ALK1 heteromeric complex, allowing for the phosphorylation of R-SMADS and potentiating subsequent nuclear-cytoplasmic shuttling to regulate gene expression [71]. ALK1 primarily mediates the effects of BMP9, but also TGF-β [72]. BMP9 and other BMP ligands serve as quiescence factors protecting cells from excessive proliferation and apoptosis [73].

About 54 different missense mutations have been reported in patients with PAH or HHT [74]. Mutations in ALK1 have been associated with HHT type 2 (HHT2), while ENG is mutated in HHT type 1 [75]. Mutations in ALK1 impairs the BMPR2/TGF-β signaling pathways, resulting in excessive proliferation and apoptosis resistance [76,77,78,79]. Eventually, this may lead to structural and functional alterations of the pulmonary vascular tree and promote PAH [80].

Interestingly, ALK1 knock-out mice develop spontaneous signs of PH within 10 weeks and show a significant increase in right ventricular systolic pressure (RVSP), RV hypertrophy, and vascular remodeling [81]. Within 18 weeks, the ALK1-deficient mice showed strong lung vascular remodeling, severe obstruction in vessels, rarefaction of peripheral arteries, and an increase in the pulmonary vascular resistance, which is associated with an increase in the PAP [81]. Mechanistically, the authors showed that the higher production of reactive oxygen species (ROS) via direct inhibition of eNOS-dependent pathway induced intrinsic defects in vasomotor tone in ALK1 knock-out mice and had little effects on the fibrosis level [81].

### 6.5. SMAD9

The SMAD9 gene encodes for SMAD8 protein, a downstream mediator of the BMP signaling alongside SMAD1 and SMAD5 [30]. Less common in PAH, mutations in the SMAD9 alter the BMP signaling pathway [30]. After the BMP ligands bind to BMPR2, BMPR2 phosphorylates various proteins, including BMPR1, ALK1, ALK2, ALK3, or ALK6 [30]. The ligand-receptor complex phosphorylates SMAD1/5/9, which subsequently phosphorylates SMAD4 [30]. Activated SMADs translocate from the cytoplasm into the nucleus and regulate the expression of target genes [31]. SMAD1/5 can compensate for the loss of SMAD4 function by phosphorylating common downstream effectors and mediating the BMP-induced canonical signaling [31]. Interestingly, the SMAD4 pathway is involved in the microRNA (miRNA) induction and maturation in a post-transcriptional manner [82]. Several studies suggested that SMAD9 mutations may trigger the development of PAH by affecting miR-processing but not canonical BMP signaling [83]. Heterozygous or nonsense SMAD9 mutations have little effect on the BMP signaling due to some compensatory mechanisms mediated by SMAD1/5 [84]. Alteration of the miR-induction and maturation downregulates two specific miRNAs in pulmonary vascular cells: miR-21 and miR-27a [83]. In PAH, the levels of miR-21 and miR-27a were both significantly lower [83]. In PAH, the downregulation of miR-21 and miR-27a is associated with a hyperproliferative phenotype of the vascular cells located in pulmonary arteries. Interestingly, overexpression of miR-21 or miR-27a significantly reduced the proliferation rates of PASMC and PAEC [83]. Overexpression of Wild-type (WT) SMAD9 in PAEC carrying a SMAD9-R294X mutation corrects miR-processing, normalizes the BMP responsiveness, and reverses the hyperproliferative state of PASMC and PAEC [83]. In addition, SMAD9 loss-of-function mutations inhibit microRNA maturation, which may explain the dysregulation of microRNAs regulated in this manner may play an essential role in PAH [82,83].

Consistently, SMAD9 knock-out mice showed complete abrogation of miR-induction while the canonical signaling was only reduced by a third. Smad8 is critical for miR processing, and the loss of Smad8 was not entirely compensated by Smad1 and Smad5 in regards to miR processing and thus represented a non-redundant function of Smad8 [83]. As mutations within the SMAD4 gene prevent miRNA maturation, this may suggest that the SMAD4-regulated miRNAs are critical for a normal phenotype. Thus, the loss of SMAD4 and subsequent maturation of microRNA might promote the pathogenesis of PAH [85]. Phosphorylation of SMAD 1/5/8 induces the recruitment of Smad4 and triggers the nuclear translocation of the complex to regulate the gene expression of genes containing B recognition element (BRE) promoters.

Knock-out mouse models for SMAD1/5 in endothelial cells and smooth muscle cells showed elevated pulmonary artery pressure, RV hypertrophy, and thickening of the pulmonary arteries. Transgenic SMAD1/5-deficient mice had increased alpha-SMA-positive distal arteries and medial wall thickness. It is important to note that only a portion of the SMAD1/5 knock-out mice showed PH phenotypes. The incomplete penetrance could be attributed to the redundancy of the SMAD1/5/8 signaling and the activation of compensatory pathways. Synergistic genetic interactions and compensatory mechanisms have been shown between SMAD1 and SMAD5 in ECs [86].

### 6.6. Eukaryotic Initiation Translation Factor 2-alpha Kinase 4 (EIF2AK4)

Eukaryotic Initiation Translation factor 2-alpha kinase 4 (EIF2AK4) belongs to a family of serine and threonine kinases and encodes a kinase named General Control Nonderepressable 2 (GCN2). GCN2 phosphorylates the alpha subunit of eukaryotic translation initiation factor-2 (EIF2S2) and decreases protein synthesis in response to amino acid starvation, hypoxia, viral infection, and specific stress response proteins. EIF2AK4 acts as a metabolic-stress sensor by detecting amino acid deficiency through binding a tRNA [87]. The role of EIF2AK4 in PAH and the underlying molecular and cellular mechanisms are currently under further investigation.

EIF2AK4 knock-out mice showed exacerbated inflammatory responses to stress, decreased autophagy, and increase oxidative stress [88]. It is still not clear if these mechanisms are responsible for intimal fibrosis and endothelial cell proliferation in pulmonary arteries in heritable pulmonary veno-occlusive disease [89]. Biallelic loss-of-function decreased the expression of Tribbles-Pseudokinase 3 and inhibited the BMP-mediated signaling [89].

EIF2AK4 gene has been identified as disease-causing in patients with pulmonary veno-occlusive disease (PVOD) and pulmonary capillary hemangiomatosis (PCH) in patients. Although homozygous mutations in EIF2AK4 are more likely to promote HPAH, there is a growing body of research that speculates that the second hit in heterozygous mutations within EIF2AK4 could also potentially lead to PAH. Since a single hit in each gene has very low penetrance, a mutation within the EIF2AK4 or BMPR2 gene could present with high penetrance and a higher likelihood of developing PAH. Christina et al. showed that nonsense mutations in EIF2AK4 in exon 8, with no other mutations in known candidate genes (BMPR2, ACVRL1, ENG), can cause PAH in an autosomal dominant manner [90].

In PAH, the occurrence of several mutations in genes identified as “drivers” gives further support to the notion that multiple genes with mutations causing PAH are more frequent than thought before. Increasing evidence shows that mutations in a specific gene usually do not potentiate PAH; however, cooperative mutations affecting multiple genes known as drivers of PAH might correlate with higher penetrance and likelihood of developing PAH.

### 6.7. Caveolin-1 (Cav-1)

Caveolin-1 (Cav-1) is a significant component of caveolae membranes and is highly expressed in ECs. It is involved in the regulation of several signaling pathways (ex: TGFβ, NO signaling) and biological processes, including proliferation, cell growth, migration, differentiation, apoptosis, and endocytosis [91]. It acts as a tumor suppressor protein at the early stages of cancer by inhibiting several mitogenic pathways [91,92]. In vascular cells, caveolae regulate the signaling of tyrosine kinase receptors and serine-threonine receptors, including BMPR2 [93,94,95,96].

Previous studies found that Cav-1 levels are significantly lower in the lung tissue of PAH patients [97]. As the BMP receptors are located in caveolae and endocytosis through clathrin-coated pits, CAV-1 is thought to regulate the SMAD-dependent signaling and vascular proliferation [98]. In 2005, Nohe et al. showed that binding of BMP-2 to its receptors released caveolin-1 and activated the SMAD signaling in COS-7 cells [99]. Concomitantly, disruption of caveolae blocked the initiation of the SMAD signaling upon BMP2 stimulation. Several studies showed that CAV-1 loss-of-function mutations might have important implications in the PAH pathogenesis. Indeed, it has been previously demonstrated that Cav-1-deficient mice (Cav-1^−/−^) exhibit higher eNOS activity and develop PAH through PKG nitration [68]. Interestingly, pharmacological inhibition of eNOS by NG-nitro-l-arginine methyl ester (L-NAME) treatment attenuated the PAP, RV hypertrophy, and prevented adverse lung remodeling in Cav-1^−/−^ mice [68]. Finally, Wunderlish et al. also found that eNOS uncoupling may trigger the development of severe cardiomyopathies in Cav-1^−/−^ mice [100].

C-Terminal mutants of Cav-1 show similar effects on eNOS by negatively regulating eNOS activity [101]. Seven residues within the Cav1 protein are essential for protein-protein interactions, and if those residues stay preserved in C-terminal mutants, the effect on eNOS inhibition is not affected [101]. Despite the similarities between human PAH and rodent models of PAH, several limitations have been raised to studying the impact of CAV1 mutations in human PAH. For example, rodent models used to study the effect of CAV1 mutations are performed in homozygous knock-outs. It is of great importance that further studies establish how the heterozygous mutations that are identified induce PAH in humans using a complementary approach between engineered heterozygous mouse model in vivo and human cells in vitro. Additionally, the protective role of the heterozygous mutations in the CAV1 gene toward the alveoli should be further investigated to better understand how they might limit the alveolar changes in patients with CAV1 mutations [102]. Finally, accumulating evidence suggests that endothelial dysfunction may mediate the adverse cardiopulmonary phenotype in Cav-1 knock-out mice and further highlights the importance of caveolae in the homeostasis of the pulmonary vasculature.

### 6.8. KCNK3

The Potassium Channel Subfamily K Member 3 (KCNK3) gene encodes a pH-sensitive two-pore domain K+ (K2P) channel expressed on PASMCs. KCNK3 dimerizes with other KCKN3 subunits or acid-sensitive KCNK9 channels. Mutation in the KCNK3 gene is responsible for the first channelopathy identified in PAH. A total of 10 different loss-of-function mutations in the KCKN3 gene have been identified in patients with PAH [103]. Mutation within the KCNK3 gene inhibits the function of the potassium channel and downregulates the expression of KCKN3 in PASMCs in vitro, inducing membrane depolarization and subsequent opening of voltage-gated calcium channels [104]. Altogether, the increase in free cytosolic calcium concentration in PASMCs promotes vasoconstriction, proliferation, migration, and resistance to apoptosis, which may increase pulmonary vascular remodeling.

Previous in vitro studies showed that decreased activity and expression of K+ channels in human PASMCs are associated with the development of IPAH and hypoxia-induced PH [105,106,107]. The up-regulation of the KCKN3 channel results in hyperpolarization of the membrane and results in calcium gated-voltage channel closure and, thus, decreases the free calcium concentration, which potentiates vasodilation in newborn piglet models of PAH [108]. In PASMCs, hypoxia inhibited the expression of KCNK3 and was associated with enhanced cell proliferation [109]. Similarly, pharmacological activation of KCNK3 using the ONO-RS-082 compound inhibited cell proliferation and STAT3 activation [109]. Selective activation of these channels by pharmacological intervention attenuates PAH in animal models [109]. The downregulation or loss of K+ channels is associated with lower apoptosis in human and rat PASMCs [110]. While there is strong evidence that the loss of KCNK3 may contribute to the development of PAH in humans, KCNK3 deletion in mice does not affect hypoxia-induced vasoconstriction [111]. These results suggested that KCNK3-deficient mice may not be the ideal model to mimic the pathogenesis mechanisms occurring in humans.

### 6.9. T-Box Transcription Factor 4 (TBX4)

T-box transcription factor 4 (TBX4) belongs to the T-box gene family. T-box genes are transcription factors that help with establishing cell type-specific gene expression patterns through epigenetic mechanisms by interacting with histone acetylases and deacetylases. It is expressed in the allantois, hindlimb, proctodeum, and throughout the mesenchyme of the developing lung and trachea [112,113]. It is critical for the development of the respiratory tract and lung branching during embryogenesis through its interaction with Fgf10 [113]. Deletions of chromosome 17q23.2, which includes the TBX4 gene, have been associated with the development of small patella syndrome (SPS) and PAH in patients [114].

While the TBX4 mutations are more frequent in children with PAH, this correlation is reversed in adult patients. The loss of TBX4 resulted in fewer foci of Fgf10 expression in the conditional Tbx4 null mice [113]. A decreased in Tbx4 expression affects mesenchymal Fgf10 expression and expression of BMP4 in the epithelium [113]. In physiological conditions, TBX4 expression leads to the optimal activity of Fgf10 and BMP4 expression, which potentiates the BMP signaling and prevents the development of PAH [113].

Although TBX4 controls multiple processes during the development of the respiratory system, its role in the pulmonary vascular tree is still relatively unknown. Tbx4-deficient embryo’s ECs showed abnormal vasculogenesis, a process by which new blood vessels form by sprouting from pre-existing vessels in the allantois [115]. The balance between the BMPR2 and TGF-β regulates various biological processes such as differentiation or proliferation. Although the TGF-β pathway promotes a proliferative state, the BMP pathway is implicated in differentiation. These pathways are highly interlinked in the development of PAH. By downregulating the FGF10/BMP4 axis, the loss of TBX4 function may dysregulate the BMP/TGFβ/SMAD pathway and trigger the development of PAH by promoting cell proliferation [114]. Further investigation is needed to decipher the signaling pathway and identify the downstream effectors of TBX4, which could potentially offer novel therapeutic candidates and offer alternative approaches against PAH.

### 6.10. Ten-Eleven Translocation 2 (TET2)

DNA methylation has been shown to increase methylation of several genes disease progression, and excessive DNA methylation inhibits transcription [116]. TET2 protein is a methylcytosine dioxygenase that catalyzes the conversion of methylcytosine to 5-hydroxymethylcytosine. DNA methyltransferases add methyl groups to DNA, and TET2 removes methyl groups from cytosine nucleotides in the DNA [117] preclinical studies have suggested that epigenetic dysregulation may trigger the development of PAH. Somatic mutations in TET2 have been found in cardiovascular diseases with clonal hematopoiesis, inflammation, and vascular remodeling [118].

Potus et al., evaluated gene-specific TET2 exome sequencing datasets from one of the largest PAH cohorts, including 2572 patients using the PAH Biobank. The novelty of this Biobank arises from also comprising individuals with non-European ancestors. Within this cohort, gene-specific rare variant association tests were performed using 1832 unrelated European patients with PAH and 7509 non-Finnish European subjects from the Genome Aggregation Database (gnomAD) as control subjects. The authors also used an independent cohort of 140 patients to measure TET2 expression in peripheral blood mononuclear cells. In this study, the authors identified nine unique germline variants in eight patients and three somatic variants in three patients. Importantly, all mutations were unique, never previously reported as “PAH gene”. Analysis of the two-dimensional structure of the protein revealed that the six deleterious variants (germline and somatic) are located within the catalytic (TET/J-binding protein methylcytosine dioxygenase activity) domain. TET2 variant carriers exhibited increased overall inflammation compared with age and sex-matched PAH patients with no mutations [26]. Potus et al. found increased expression of 30 proinflammatory cytokines in TET2 variant carriers. For example, IL-1b expression was increased in 70% of patients with TET2 mutations compared to PAH patients with no mutations. In peripheral blood mononuclear cells, TET2 gene expression was decreased by 86% in systemic sclerosis (SSc)-PAH patients and decreased by 86.7% in iPAH patients compared relatively to healthy controls [118]. The authors developed conditional, hematopoietic heterozygous (Tet2^+/−^) and homozygous (Tet2^−/−^) knockout models to mimic TET2 deleterious variants in human patients. In vivo studies conducted by Potus et al. showed that hematopoietic TET2-knockout mice spontaneously developed a PH-like phenotype and notably manifested a profound loss of the pulmonary microvasculature. TET2 depletion significantly increased RVSP, total pulmonary resistance, arterial elastance, adverse pulmonary vascular remodeling, decreased perfusion of distal pulmonary arteries, and obliteration of the microvasculature. PH development was also associated with increased inflammation in the lungs of TET2 knock-out mice, as demonstrated by macrophage accumulation and dysregulation of 61 inflammatory markers, with 59 inflammatory markers being upregulated [118]. In this study, Potus et al. also showed that IL-1B blockade as anti-inflammatory therapy reverses the PH phenotype in TET2^−/−^ mice. IL1-β antibody treatment improved PH hemodynamics and phenotype parameters in TET2^−/−^ mice, evidenced as decreased Fulton index, increased pulmonary artery acceleration time and decreased RVSP, mean pulmonary arterial pressure (mPAP), and total pulmonary resistance (TPR) index.

Although the link between PAH and TET2 mutation variants needs to be studied further, this study identified for the first time new mutations within the TET2 gene using one of the largest cohorts available to date. With convincing evidence, the authors further demonstrated that TET2 mutations or TET2 deficiency might potentiate PAH through an inflammation-dependent mechanism. Additionally, this study also suggests that inflammation associated with aging might potentially act as a second hit in PAH patients carrying TET2 deleterious variants.

### 6.11. Other Genetic Determinants of Pulmonary Hypertension

Additional rare sequence variations contribute to the genetic and molecular profile of PAH phenotype. In a recent whole-genome sequencing (WGS) study by Graf et al. comprising 1048 PAH cases and 6385 controls, the authors first removed samples with deleterious mutations that were previously described as “PAH genes”, including BMPR2, ACVRL1, ENG, KCNK3, SMAD9, and TBX4 to further increase the statistical power of the analysis. Several previously unidentified genes (ATP13A3, AQP1, and SOX17) have been shown to be significantly upregulated in PAH cohort studies [56].

ATP13A3 showed three heterozygous frameshift variants, two top gained, two splice region variants, which were predicted to lead to a loss-of-ATPase catalytic activity. Additionally, the authors identified four heterozygous pathogenic missense variants among the PAH cases and two variants near the conversed ATPase catalytic site, which is predicted to destabilize the conformation of the catalytic domain [56]. ATP13A3 is a poorly understood P-type ATPase of the P5 family, and little is known regarding the function of ATP13A3. Even though the precise mechanism of ATP13A3 is unknown, it is thought to play a role in polyamine transport [119]. Interestingly, RNA sequencing datasets showed that ATP13A3 is highly expressed in PASMC and PAEC [56]. Inhibition of ATP13A3 in vitro decreased PAEC proliferation while increasing apoptosis. This finding is consistent with previous studies showing that endothelial apoptosis is a major initiation factor for the onset of PAH [120]. However, the role of ATP13A3 in vascular cells and its function surrounding BMP signaling remain largely unknown.

Rare variants of the AQP1 genes were identified in PAH patients. The AQP1 gene encodes aquaporin-1 and is described as the water channel. The p.Arg195Trp variant was identified in five PAH cases and affected the hydrophilic face of the pore [56]. The arginine at the 195 position helps define the constriction region of the AQP1 pore structure and is conserved across aquaporins [121]. Aquaporin-1 belongs to a family of membrane channel proteins that facilitate water transport in response to osmotic pressures, and AQP1 is thought to promote EC migration and angiogenesis [121]. AQP1 inhibition in PASMCs attenuates hypoxia-induced PH in mice [122]. However, further studies are needed to define the role of AQP1 in pulmonary vascular cells and determine the functional impact of the AQP1 variants on water transport. The study by Rhodes et al. also concluded that unaffected AQP1 variant carriers showed reduced PAH penetrance.

A total of four nonsense variants of the SOX17 gene were reported. Premature truncating variants (PTV) are predicted to lead to a loss of the β-catenin binding region. In addition, six missense variants are predicted to disrupt the interaction with Oct4 and beta-Catenin. SOX17 mechanism of action remains poorly understood, and how SOX17 causes PAH still needs to be confirmed. SOX17 encodes the sex-determining region Y (SRY)-box containing transcription factor 17 and is implicated in angiogenesis [123] and arteriovenous differentiation [124]. Previous studies showed that conditional deletion of SOX17 in mesenchymal progenitors leads to impaired lung microvessel formation [125]. The demonstration of SOX17 p.Y137*PTV with early-onset PAH provides a causal link for the role of these variants in PAH [56]. A study on affected offsprings revealed that the coexistence of patent ductus arteriosus in the index case and an atrial septal defect (ASD) might suggest an association with congenital heart disease [56]. Even though small ASDs are not uncommon in IPAH, more detailed phenotyping of SOX17 mutation carriers is needed to determine whether the presence of ASDs are more common in carriers of these mutations (Table 1).

## 7. Precision Medicine

Although conventional therapies reduce the symptoms of PAH by leveraging three distinct pathways involved in the vasoconstriction and vasodilation imbalance, none of the approved therapies have shown satisfactory results in reversing PAH. Accumulating clinical evidence suggests that the shortcoming of current treatments may be due to the multifactorial nature of PAH and the genetic variations among patients [126,127,128,129,130,131].

Precision medicine is a novel strategy that focuses on individual differences both at the molecular level and through state-of-the-art panomics profiling, in concert with high-level data derived from a patient’s lifestyle, medical examinations, and environmental exposures to better determine a person’s health or disease phenotype [132].

**Figure 2:**
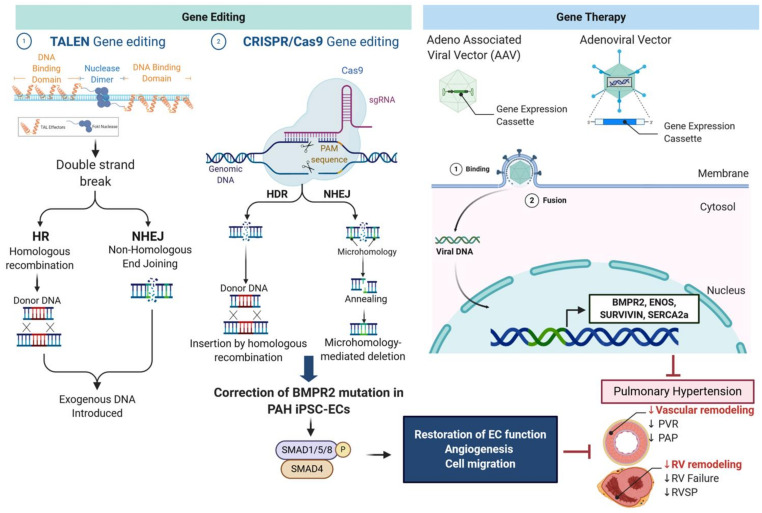
Therapeutic potential of genomic medicines and gene therapy in PAH. Nowadays, gene-based therapies are considered as transformative medicines. Among the most common, gene editing and gene replacement therapy are two powerful tools used to precisely edit genomes or deliver a working copy of the missing or defective. Gene editing-strategies include TALEN and CRISPR technology. Both rely on site-specific cleavage and subsequent gene editing through the cell’s DNA repair machinery by Homologous Recombination (HR) or Non-Homologous End Joining (NHEJ). Importantly, the gRNA provides the DNA repair template and allows the replacement of an existing segment. Gene therapy aims to restore BMPR2, eNOS, Survivin, and SERCA2a expression and ultimately inhibits the vascular and cardiac remodeling while improving the hemodynamic parameters in rodent models of PAH. Adeno-associated viruses (AAV); Bone morphogenetic protein receptor type 2 (BMPR2); CRISPR associated protein 9 (Cas9); Clustered Regularly Interspaced Short Palindromic Repeats (CRISPR); single guide RNA (sgRNA); Sarcoplasmic/endoplasmic reticulum Ca2+ ATPase 2a (SERCA2a); Transcription activator-like effector nucleases (TALEN).

Multi-Omics approaches are high-throughput unbiased technologies and are commonly used to better characterize patients by deeply analyzing the genomics, transcriptomics, epigenomics, proteomics, and metabolomics signatures [133], and help to define the prognosis and predict drug responsiveness [133]. The generation of individualized maps provides valuable information to select the best therapeutic strategy for each patient and improve the outcome [134]. Moreover, the identification of new single nucleotide polymorphisms arising from omics data has contributed to the development and design of new therapeutic genomic medicines as well as innovative therapeutic delivery methods to overcome genetic disorders. Gene editing-based strategy and gene replacement therapy represent two powerful tools to precisely edit genomes and deliver a working copy of the missing or defective gene using a vector, respectively [135,136]. Both strategies have shown remarkable results against respiratory diseases, including PAH and pulmonary fibrosis [26,137] (Figure 2).

### 7.1. Genome-Editing Technologies

Currently, two core gene-editing technologies are derived from bacteria and have shown increasing interest among the scientific community in the prevention and treatment of human diseases: transcription activator-like effector nucleases (TALEN) and Clustered Regularly Interspaced Short Palindromic Repeats (CRISPR) [136,138,139]. Fundamentally, these two gene-editing technologies rely on site-specific cleavage and subsequent gene editing through homologous recombination (HR) or non-homologous end-joining (NHEJ). HR plays a critical role in gene editing, as it provides a template to repair the damage [139]. In the absence of HR, CRISPR, or TALEN showed limited efficacy and are only capable of silencing or truncating a gene.

TALEN are widely used as a precise and efficient gene-editing technology in live cells [139]. Artificial restriction enzymes are generated by fusing a transcription activator-like effector DNA-binding domain to a DNA cleavage domain [139]. By combining a TAL Recognition sequence with a DNA cleavage domain, the engineered restriction enzymes can specifically recognize any desired DNA sequence [139]. The insertion of specific sequences induces double-stranded breaks at a particular location on the DNA sequence and activates repair mechanisms [139]. The Clustered Regularly Interspaced Short Palindromic Repeats (CRISPR) technology, also called CRISPR, refers to a recently developed gene-editing technology that can target any location in the genome and edit the DNA sequence with high specificity [139]. It relies on the combination of two types of molecules: a CRISPR-associated endonuclease (Cas protein) and a target-specific single guide RNA (gRNA or sgRNA) [139]. The gRNA is a short synthetic RNA that specifically recognizes the DNA sequence of interest and confers target sequence specificity to the CRISPR-Cas9 system [139]. It is composed of a scaffold sequence necessary for Cas-binding and a ~20 nucleotide spacer that defines the genomic target to be edited [139]. Importantly, the gRNA provides the desired sequence that serves as a DNA repair template [139]. Similar to the TALEN technology, CRISPR-Cas9 relies on the cell’s DNA repair machinery to edit the genome by replacing an existing segment with a customized DNA sequence [139].

In PAH, many genetic mutations have been identified and found to predispose to developing PAH. Therefore, the research and development of new genomic medicines using gene-editing technologies such as CRISPR-Cas9 or TALEN have the potential to correct previously identified deleterious mutations (Figure 2). For example, an autosomal dominant mutation within the BMPR2 gene is detected in 75% of HPAH cases and is responsible for the haploinsufficiency or loss of function of BMPR2. It has been identified as the major genetic cause of PAH [35]. Thus, gene editing the bone marrow-derived stem cells for BMPR2 mutations before transplantation could provide a powerful strategy for curing PAH [140]. In 2017, Gu et al. used induced pluripotent stem cell-derived endothelial cells (iPSC-ECs) from three families with unaffected mutation carriers (UMC), familial PAH (FPAH) patients, and gender-matched controls to investigate this variation [140]. Interestingly, the authors found an increase in BMPR2 activators and a reduction in inhibitors in UMC iPSC-ECs [140]. Using the CRISPR-Cas9 system to correct the *BMPR2* mutation in PAH iPSC-ECs, the authors demonstrated that correcting the BMPR2 mutation successfully restores the BMPR2 signaling, EC function, and normalized angiogenesis and cell migration upon BMP4 stimulation [140]. However, this technology needs to be further investigated to identify possible off-target genes and prevent genomic instability or terminal differentiation of stem cells. Currently, editing by homologous recombination takes months, and a deep understanding of the signaling factors is critical to maintaining the stem cell phenotype for these technologies to be rolled out to future clinical applications.

### 7.2. Adenoviral and Adeno-Associated Virus Serotype 1 (AAV1)-Mediated Gene Transfer

Over the past decades, extensive research has been made in the field of gene therapy to optimize cell specificity and decrease immunogenicity while enhancing the transduction efficacy [141,142,143]. AAV-based gene transfer has shown promising results with high and long-term transduction in animals with limited immunogenicity [141,142,144,145,146] (Figure 2). Recent optimization in the vector designing and engineering have significantly improved the in vivo tissue-tropisms of the AAV serotype vectors and reduce the off-target effects [147,148,149].

### 7.3. BMPR2

Restoration of BMPR2 expression using gene therapy has shown promising results in animal models and under research settings. Intratracheal aerosolized delivery of adenoviral BMPR2 gene transfer reduced PASMC and PAEC proliferation in experimental PAH animals. It also significantly reduced the vascular muscularization in the MCT-induced-PAH model and hypoxia-induced PH by up to 40% [150,151]. Reynolds and colleagues showed that intratracheal delivery of an adenoviral vector carrying the BMPR2 gene successfully up-regulated the expression of BMPR2 in the pulmonary vasculature and restored the SMAD-1/5/8 while reducing the SMAD-2/3 signaling [150]. The same group demonstrated that lung-targeted adenovirus-mediated BMPR2 gene therapy significantly reduced the RV hypertrophy as well as the PAP in the MCT-induced PAH model in rats [151]. These findings support the therapeutic benefits of BMPR2 gene therapy by potentiating the switch from the TGF-β/SMAD-2/3 signaling to the BMPR2/SMAD-1/5/8 signaling. In 2016, Harper et al. demonstrated that adenoviral gene delivery of BMPR2 inhibited MCT-induced PAH, as illustrated by the reduced RV hypertrophy, RV systolic pressure, mean PAP [152]. At the molecular level, the authors found that gene delivery of BMPR2 increased the activation of the Phosphoinositide-3 kinase (PI3K) pathway and decreased the phosphorylated-p38mitogen-activated protein kinase (P38-MAPK) signaling in vivo.

However, another study showed contradicting results. McMurtry and collaborators demonstrated that the overexpression of the BMPR2 receptor using a nebulized adenovirus encoding for human BMPR2 (Ad.hBMPR2) did not improve MCT-induced PAH in rats [153]. Restoring BMPR2 expression in MCT-induced PAH did not attenuate the PAP, PVR, and did not improve the cardiac index and RV hypertrophy [153]. The transduction efficiency of Ad.hBMPR2 was high, as evidenced by laser-capture microdissection, and therefore under-dosing was unlikely to account for their negative results. Nevertheless, adenovirus vectors can trigger inflammation in transduced tissues and potentially lead to a rapid loss of transgene and vector. Vector immunogenicity of the different AAV serotypes and adenovirus remains a major limitation for even the new generation of adenovirus vectors, particularly when high titers are required. Adenovirus vector-induced innate immune responses may counteract the beneficial effect mediated by the transgene overexpression. The recent development of several recombinant AAV serotypes has significantly improved the tissue tropism and improved transduction efficiency in smooth muscle cells and endothelial cells while reducing the vector immunogenicity. Lastly, increasing evidence shows that the pathogenesis of MCT-induced PAH may not be identical to human PAH, suggesting that the failure of BMPR2 gene therapy might be due to model-specific factors [153].

### 7.4. Survivin

Adenoviral vector encoding a dominant-negative Survivin has also proven impressive results in vivo [154]. Survivin is an “inhibitor of apoptosis” protein expressed in the pulmonary arteries of patients with PAH. Inhaled adenoviral gene therapy of a phosphorylation deficient-Survivin mutant, with dominant-negative properties, reversed MCT-induced PAH and prolonged the survival by 25% in animal models [154]. The dominant-negative mutant also lowered PVR, RV hypertrophy, and the medial muscularization in pulmonary arteries [154]. The authors also found that Survivin inhibition using a dominant-negative construct also induced PASMC apoptosis, decreased proliferation, and depolarized mitochondria, which cause efflux of cytochrome C in the cytoplasm and translocation of apoptosis-inducing factor into the nucleus. Inhibition of Survivin in humans could be a novel therapeutic option [154].

### 7.5. eNOS

Similarly, lung delivery of an adenovirus-encoding constitutive eNOS in rats increased eNOS expression or activity and reduced hypoxia-induced pulmonary vasoconstriction [155]. Another study conducted by Zhang et al. showed that intra-tracheal delivery of recombinant adenovirus encoding the eNOS gene attenuated flow-induced PH in rabbits [156]. In this model, external jugular vein ligation was performed in rabbits to create an arteriovenous shunt and mimic the chronically increased pulmonary artery flow-state of a congenital heart defect. Then, three months after the shunt operation, the data showed a significant reduction in mPAP and PVR in rabbits who received intratracheal instillation of adenovirus with CMV promoter (Ad.CMV) encoding eNOS compared with Ad.CMV-Null-treated rabbits [156]. Immunohistochemical staining showed that eNOS expression was mainly detected in the vascular endothelium, suggesting that the instillation of recombinant adenoviruses encoding for a gene with vasodilatory properties may be used as a selective therapy to attenuate hypoxic pulmonary vasoconstriction. Lastly, stem cell therapy showed to be effective in attenuating the RV impairments associated with PAH. A recent study showed that implantation of eNOS gene-transduced mesenchymal stem cells (MSC) had shown promising results in monocrotaline (MCT)-induced PAH rats [157]. Implantation of MSC and eNOS significantly lower the RVSP, the RV hypertrophy induced by MCT, and increased the survival time [157]. Regeneration of endothelial cells increased the vascular beds and, therefore, the secretion of NO from these cells. Restoration of eNOS activity may potentiate the vasodilation of pulmonary arteries and reduce PH [157]. The results showed that intravenous implantation of MSCs overexpressing eNOS might provide a novel insight into RV impairment caused by PAH [157].

However, inhaled eNOS adenoviral gene therapy showed important limitations in clinical and pre-clinical studies due to the vector-induced inflammation and toxicity. In animal models of PAH, cellular and humoral immune responses produced immunoreactivity against the first generation adenoviral vectors. Furthermore, adenoviral eNOS gene therapy only induced transient effects lasting less than 14 days with a single administration [158]. However, the repetitive administration of adenovirus-mediated gene transfer for long-term expression is due to the adenoviral vectors’ toxicity with high titers and strong innate immune response in mediating an acute inflammatory reaction [159]. Gene therapy approaches using MSC overexpressing genes of interest such as eNOS have also been less effective than expected. While MSCs have the pluripotent ability to become endothelial cells and restore normal NO functioning, the overexpression of eNOS can lead to apoptosis and delivery of MSCs can potentially lead to pulmonary embolisms [157].

### 7.6. Voltage-Gated K^+^ Channels

Type voltage-gated potassium (Kv) channels gene therapy may also be beneficial in preventing the progression of PAH by restoring the balance of cytosolic Calcium. Contractility and proliferation of PASMCs are regulated by the intracellular concentration in Calcium, which is determined by membrane potential. Vascular cells isolated from PAH patients and rodent models of PAH show more depolarization of the membrane due to decreased expression of voltage-gated potassium channels. This potassium channelopathy leads to membrane depolarization and calcium overload, which induce PASMCs contraction. Restoration of Kv channel expression by aerosolized gene therapy using adenovirus expressing Kv2.1 or Kv1.5 reduces PVR and restores RV hypertrophy, and enhances apoptosis in human PASMCs [160,161].

### 7.7. Growth Factor Gene Therapy

Growth factor gene therapy to overexpress the vascular endothelial growth factor (VEGF-A) using cell-based gene transfer prevents MCT-induced PAH. Using a cell-based gene transfer approach, smooth muscle cells overexpressing VEGF-A (SMC-VEGF-A) were injected into the internal jugular vein of MCT-rats [162]. The authors found that MCT-rats treated with SMC-VEGF-A had reduced RV pressure and hypertrophy. Even four weeks after the gene transfer, high VEGF expression was still detectable in the pulmonary tissue of the treated rats. This lends support to the survival of the transfected cells and persistent gene expression in pulmonary vasculature [162].

Similarly, Human Growth Factor (HGF) therapy has shown promising results in MCT-induced PAH in rats [163]. Ono et al. used a plasmid encoding human HGF cDNA with the hemagglutination virus of Japan (HVJ) as a delivery vehicle [163]. The HVJ-Liposome complex was inserted via midline laparotomy into the left lobe of the liver. The HVJ-Liposome induced overexpression of human HGF in the liver and reduced medial wall thickening of pulmonary arteries [163]. The data gathered by Ono et al. suggest that, compared with direct gene transfection to the lung, the transfection to the liver needs higher doses due to the hemodynamic effect and suggested that a sophisticated method of gene transfection is required to enable long term transduction and maintain adequate plasma concentrations without side effects [163].

VEGF-A and HGF may have protective effects against pulmonary vascular remodeling and may be potential candidates for gene therapy. Growth Factor gene therapy is still under investigation to understand the mechanisms by which they protect from vascular remodeling. These studies are preliminary and were only conducted in rats using the MCT-induced PAH model.

### 7.8. Sarcoplasmic/Endoplasmic Reticulum Ca2+ ATPase 2a (SERCA2a)

Early studies have identified a significant downregulation in SERCA2a protein and mRNA levels in lung samples from patients with PAH [144,145,146,164,165]. Therefore, our group has conducted gene transfer therapy to restore SERCA2a expression using AAV1-mediated gene therapy in respiratory diseases, including PAH, and using various distinct pathological models in rodents [144,145,146,164,165]. Interestingly, intratracheal inhalation of aerosolized AAV1 carrying the human SERCA2a gene (AAV1.SERCA2a) inhibited PAH in the MCT-induced PAH rat model and the chronic post-capillary PH model in Yorkshire pigs by decreasing the PAP, vascular remodeling, and RV hypertrophy compared to controls [144,145,146,164,165]. Similarly, nebulized AAV1.SERCA2a gene therapy significantly reduced pulmonary vein banding-induced PH in Yukatan miniature swine and increased long-term survival [146]. In PASMCs, SERCA2a overexpression significantly decreased proliferation and migration by inhibiting the nuclear factor of activated T-cells (NFAT)/STAT3 pathway [145]. In PAECs, SERCA2a overexpression also increased eNOS expression [145].

Several gene therapy treatments aim to reduce or reverse the PAH pathophysiology without affecting the systemic circulation. As of today, gene delivery via intratracheal delivery of aerosolized adenovirus or AAV1 carrying wild type genes has shown promising results as they decrease the risks associated with intravenous injections such as nuclease degradation and show limited penetration through the endothelial barrier. The use of gene delivery via inhalation depends crucially on the development of advanced gene vectors. The gene vectors must be able to protect the plasmid or sequence from nuclease-mediated degradation and provide a specific tissue tropism. Clinical trials with AAV-vectors have shown little effect due to the immunogenic responses and the production of neutralizing antibodies that limit their chronic and repeated administration [166]. A better understanding of the underlying mechanisms may provide critical insights into the prevention of the gene transfer-associated inflammatory response and may be paramount before launching further clinical trials. Combination therapy to counteract the pro-inflammatory response may provide a better therapeutic option. However, such therapies still need to be evaluated and further investigated to propel viral therapy into the mainstream paradigm for PAH.

### 7.9. Nanoparticles-Mediated Therapy

Nanoparticles (NP) are ultrafine particles described by a diameter between 0.1 and 100 nm that can be utilized to package genetic materials or synthetic treatments. These NPs can be precisely engineered to specific sizes and geometries to enable enhanced cellular entry and controlled release of treatments. NPs have been primarily used in cancer treatments due to their ability to embed passively in tumor tissues by extravasating through leaky vasculature [167]. Few different platforms for NPs can be utilized for the delivery of exogenous genes to specific tissues. Liposomes prove advantageous for the delivery of genetic delivery through the incorporation of cationic lipids. Polylactide-Co-Glycolide (PGLA) can also incorporate cationic polymers for the delivery of genetic material [167].

NF-kB is a transcription factor that regulates many inflammatory cytokines such as IL-6 and TNF-alpha, as observed in PAH. NPs packaged with a NF-κB decoy were studied using polyethylene glycol (PEG)- poly(lactic-co-glycolic acid) (PLGA) polymer to inhibit the binding of NF-κB to the promoter. In mice, intravenous administration of NPs showed effective and long-lasting release of NF-κB decoy that lasted close to a month in the systemic circulation. NF-kB decoy NPs significantly reduced RV systolic pressure, RV hypertrophy, and reduction in muscularized pulmonary arteries in MCT-induced mice [168]. NPs were also used to deliver an antisense oligonucleotide against microRNA-145. Higher levels of microRNA-145 have been identified in patients with PAH and have been associated with the development of vascular remodeling [169]. NPs encapsulated with cationic lipids with the oligonucleotide against miR-145 were delivered to Sugen- Hypoxia (SuHx) mice. AntimiR-145 significantly decreased the expression of microRNA-145 by 50% and reduced the media wall thickness [170].

Additional studies have shown that nanoparticle therapy may be effective for patients currently on vasodilators such as prostacyclin or phosphodiesterase 5 (PDE5) inhibitors. Because intravenous administration of these drugs causes adverse effects such as headaches, hypotension, and Cather-related infection, nanoparticle-mediated Drug Delivery Systems (Nano-DDS) may circumvent repeated administration of vasodilators by allowing more efficient drug delivery mechanisms and uptake by targeted tissues [171]. The intratracheal delivery of aerosolized beraprost NPs to the lungs of MCT-induced rats reduced RV pressure, RV hypertrophy, pulmonary artery muscularization, and vascular remodeling with a single intratracheal administration. Importantly, the survival rate increased to 65% following the administration of NP-based beraprost, compared to 27.8% in disease controls.

### 7.10. Conventional and Combination Therapies

Conventional therapies leverage the imbalance between vasoconstriction and vasodilation to manage symptoms such as hypertension associated with PAH. The drugs currently on the market, when combined, provide a better response than monotherapy. Epoprostenol, currently used in PAH patients, is a synthetic analog of prostacyclin, potentiating vasodilation and attenuating proliferation of PASMCs and PAECs. When combined with sildenafil (PDE-5 inhibitor), increased cyclic guanosine monophosphate (cGMP) concentrations have been reported, which allow for blood vessel relaxation and widening [172].

Combination therapies using epoprostenol and sildenafil in a randomized control trial showed better results in the 6-min walk distance (6MWD) test, while longer time was reported in patients treated with epoprostenol alone. Combination therapies also improved the hemodynamic parameters and exercise capacity in a small subset of the patients [173]. Similarly, in a STEP trial (STEP: Safety and pilot efficacy Trial in combination with bosentan for Evaluation in Pulmonary arterial hypertension), inhaled iloprost (a synthetic analog of prostacyclin PGI2) in combination with bosentan (endothelin-1 receptor antagonist) dilates systemic and pulmonary arterial vascular beds and allows for dilation of pulmonary blood vessels. Unfortunately, the latter treatment did not improve the 6MWD test compared to controls; however, it did enhance the pulmonary hemodynamics and time to clinical worsening [174].

Combinations of therapies are currently recommended for patients with severe PAH, and excellent results have been reported with triple upfront combination therapies [175]. Indeed, a study evaluated the effects of this approach on RV function and outcome in 21 patients with severe PAH [175]. Patients (age, 44 ± 15 years) with newly diagnosed high-risk idiopathic PAH that was nonreversible by the inhalation of nitric oxide were treated upfront with a combination of ambrisentan, tadalafil, and subcutaneous treprostinil [175]. Echocardiography analysis showed decreased right-sided atrial and RV areas, improved left ventricular eccentricity index, and increased fractional area change [175]. In summary, the authors found that triple upfront combination therapy with ambrisentan, tadalafil, and subcutaneous treprostinil in severe nonreversible PAH is associated with considerable clinical and hemodynamic improvement and right-sided heart reverse remodeling with a marked decrease (67–69%) in PVR [175]. This study suggested that upfront triple combination of drugs targeting endothelin, NO, and prostacyclin pathways allows for a clinical and hemodynamic improvement in severe PAH contrary to combination therapies using only one or two of these drugs.

Considering the therapeutic potential of gene editing and gene-replacement therapy, the recent development of these technologies and the emergence of genomic medicines may offer a potential new treatment avenue for patients suffering from PAH. Unlike current conventional therapies, the combination of personalized medicine that guides health care decisions toward the most effective treatment with new genomic medicines may represent an effective therapeutic strategy to cure PH. In addition, preventing the development of PH in patients carrying a defective gene using gene replacement therapy or gene editing based on individual variability is crucial in PH due to the complex and multifactorial nature of PAH. Further complications associated with the gender penetrance and broad age-onset may also contribute to the heterogeneity of drug responsiveness from patient to patient.

## 8. Resistance to Treatment

Vascular remodeling is associated with pro-proliferative, migratory, and anti-apoptotic phenotypes of PASMC and PAEC in PAH. Proliferating cells within the pulmonary artery walls are characterized by a cancer-like metabolic switch where mitochondrial glucose oxidation is suppressed while glycolysis is upregulated, promoting ATP production and structural changes in the pulmonary vascular wall [176]. The pyruvate dehydrogenase enzyme (PDH) plays a central role in glucose oxidation as it catalyzes the mitochondrial production of acetyl-CoA from pyruvate, the end-product of glycolysis, which in turn feeds the Krebs cycle to complete glucose oxidation. Another mechanism is through the inhibition of Sirtuin 3 (SIRT3) activity. SIRT3 is the main mitochondrial deacetylase, which activates several mitochondrial enzymes, including PDH. Additionally, other enzymes such as the pyruvate dehydrogenase kinase (PDK) inhibits the mitochondrial PDH activation by phosphorylation at serine-293. Lastly, uncoupling protein 2 (UCP2) has been shown to regulate PDH activity through a calcium-dependent mechanism. Previous studies reported increased PDK levels in the lungs from PAH patients and animal models of PAH. Moreover, the metabolic remodeling within the pulmonary vascular cells favors glycolysis over aerobic respiration [177].

The small molecule dichloroacetate (DCA) is currently being used in pre-clinical studies in animal models of PAH and early-phase clinical trials in congenital mitochondrial diseases and cancer [177,178,179,180,181,182], such as malignant brain tumors, squamous cell carcinoma of the head and neck, metastatic breast, and non-small cell lung cancer. Ex-vivo studies showed that lung perfusion of PAH lungs with DCA increased PDH activity and oxygen consumption in isolated PAH lungs. In a 4 month open-label study, DCA therapy showed a good safety profile and improved hemodynamics and functional capacity in IPAH patients, as evidenced by reduced mPAP and PVR. Inhibition of mitochondrial PDH by DCA has shown promise with variable efficacy. Given the variability in response to DCA therapy, Michelakis et al. further investigated whether the presence of loss-of-function gene variants for SIRT3 and UCP2 impair the ability of DCA to activate PDH, reverse mitochondrial suppression in human PAH. The authors determined that the variability in response to the DCA therapy in IPAH patients is associated with the presence of inactivating mutations in two genes encoding the mitochondrial proteins SIRT3 and UCP2. Low DCA efficacy was reported in IPAH patients carrying SIRT3, and UCP2 variants as loss-of-function gene variants counteract the ability of DCA to activate PDH and reverse mitochondrial suppression. The SIRT3 rs11246020 variants are caused by the substitution of a single nucleotide for another nucleotide. This point mutation results in a change of valine to isoleucine at residue 208 within the conserved catalytic deacetylase domain and induces a 34% decrease in SIRT3 activity. The UCP2 rs659366 variants affect the promoter region of the gene, resulting in a decrease in mRNA expression. These two single-nucleotide polymorphisms (SNP) are associated with decreased SIRT3 activity and UCP2 expression, and ultimately resistance to DCA treatment in PAH-patients [177].

In rodent models of PAH, DCA therapy reduces RV hypertrophy and increases cardiac output and exercise capacity in multiple pre-clinical models of PAH [183]. However, these results are not consistent in human lungs from PAH patients. Although DCA therapy does improve oxygen consumption in PAH lungs through stimulating PDH and improving glucose oxidation [177], these positive results are not observed in all PAH patient’s lungs. The beneficial effects of DCA mainly rely on the genetic profile and, more precisely, the absence of polymorphism within the SIRT3 or UCP2 genes in PAH patients [184]. In a 4-month open-label trial of 20 patients, DCA reduced PVR and increased 6 min walk distance test. However, these changes were more pronounced in patients with normal or low polymorphism scores and patients with normal SIRT3 and UCP2 function [184].

This study further raises a potential avenue for a personalized medicine approach for DCA therapy in patients with no genetic variation in the SIRT3 and UCP2 genes. Importantly, this study also highlights the imperative use of genetic testing and molecular profiling for a better characterization of PAH patients to determine the best-personalized medicine to improve treatment outcomes in PAH patients. This is especially important in multifactorial diseases such as PAH, where patients may respond to treatment differently based on the underlying etiology.

## 9. Big Data and Artificial Intelligence

Healthcare has always been a data-driven process. Data is crucial for decision-making and the provision of healthcare. With the increased digitalization of healthcare, there is an immense amount of generated data that can be leveraged to better inform decision-making and equip physicians with tools to identify potential patients at high risk. In this regard, artificial intelligence and machine learning algorithms may help to analyze large datasets and provide meaningful and actionable insights to support decision-making [185].

### 9.1. Transcriptional Profiling

Transcriptomic analysis can provide critical information towards gene expression changes in PAH and thus lead to the identification and characterization in a cell-type-specific manner of crucial drivers involved in disease progression or various biological processes. Previous methodologies used for gene expression analysis were limited by their inability to individually examine a particular cell type in a given tissue. Nowadays, singe-cell RNA sequencing (scRNA-Seq) is a powerful and insightful method for analyzing gene expression with single-cell resolution. It allows the examination of the transcriptomic profile of individual cells. Many novel genes implicated in PAH have been discovered using scRNA-Seq and have further extended our understanding of the disease.

In a small study consisting of six control lungs and three IPAH lungs obtained during transplantation surgery, endothelial cells and pericyte and smooth muscle cells showed the most dysregulated gene expression profile. In endothelial cells, up-regulation of the Roundabout guidance receptor 4 (ROBO4), APC down-regulated 1 (APCDD1), N-Deacetylase and N-sulfotransferase 1 (NSDT1), Multimerin 2 (MMRN2), Neurogenic locus notch homolog protein 4 (NOTCH4), and dedicator of cytokinesis 6 (DOCK6) levels were found. NOTCH4 inhibits endothelial cell apoptosis. DOCK6 is a guanine nucleotide exchange factor and activates Rho proteins, such as Cdc42, by catalyzing the exchange of guanosine diphosphate (GDP) for guanosine-5′-triphosphate (GTP). Interestingly, several transcription factors were elevated in IPAH endothelial cells, including SOX18, STRA13, LYL1, and ELK, which have known roles in regulating the endothelial cell phenotype and endothelial cell transcriptome [186].

Gorr et al., have identified several differentially expressed genes within the PASMCs from IPAH patients compared to control-PASMCs. The authors found that genes specifically involved in cell proliferation, mitosis, and inflammation were significantly dysregulated in PAH patients. For example, ADGRG6/GPR126, an adhesion G protein-coupled receptor (GCPR), was significantly increased in PASMCs from IPAH patients compared to control-PASMCs. Surprisingly, it has been shown that both GPR126 overexpression and knockdown increased proliferation and cAMP level in IPAH-PASMCs. However, these mechanisms need to be studied further to define the role of this pathway and elucidate their differential expression in cell-types and their contribution towards a differential effect on proliferation and apoptosis in PASMCs [187].

The contribution of epigenetic alterations has been investigated using a multi-omics integration approach in PAECs derived from a cohort of IPAH and HPAH patients and healthy controls. The authors integrated data from RNA-Seq, chromatin immunoprecipitation followed by sequencing (ChIP–seq) using H3K27ac, H3K4me1, H3K4me3 antibody, and chromatin interaction (ChIA-PET) profiling to gain further molecular insights into disease mechanisms. In PAH-PAECs, they found that the H3K27ac levels in PAECs were highly predictive of the expression change in response to endothelial signaling factors. The identification of the global regulatory network revealed that the regulatory state might potentially drive the cells towards an endothelial to mesenchymal transition. Stimulations with endothelial-specific growth factors such as Serotonin, VEGF, or TGF-β confirmed enhancer- priming, which could lead to abnormal growth, inflammation, aberrant angiogenesis, and EndMT or differentiation. Overall, this study demonstrated that integration of multiple omics data might be used for the identification of disease-specific pathways when steady-state expression analyses are limited in systems with an aberrant response to stimuli [188].

Finally, kinome-related gene regulation has also provided critical new insights into the clinical diagnosis of PAH by generating a precise molecular diagnostic model to differentiate PAH from PVOD. Neubert et al. generated, with high sensitivity and specificity, a neuronal network that differentiated pulmonary veno-occlusive disease (PVOD) from PAH samples. The authors identified two novel genes, Protein O-Mannose Kinase (SKG196, also known as POMK) and Microtubule Associated Serine/Threonine Kinase 2 (MAST2), that are increased in PAH. MAST2 is a kinase that forms complexes with cell-cell adhesion molecule protocadherin LKC (CDHR2, also known as PCLKC) and influences contact inhibition in the liver and kidney. SKG196 is a glycosylation-specific-O-mannose kinase involved in dystroglycan receptor function, which provides cellular adhesion of endothelial cells to the extracellular matrix. Given their respective properties on cell adhesion, they may promote vascular remodeling and migration of ECs [189]. Importantly, the identification of such neuronal networks is crucial for improving pre-established diagnostic algorithms, facilitating the early diagnosis of the disease as well as developing novel target-specific interventions by enabling clinical diagnostics in an elusive group of diseases.

ScRNA-seq analysis in lungs from MCT and SuHx-induced PAH model revealed widespread upregulation of the NF-kB signaling and downregulation of the interferon signaling across different cell types. SuHx non-classical monocytes and monocrotaline conventional dendritic cells showed strong activation in the NF-kB pathway. Hong et al. showed differentially expressed genes whose differential expression was model and cell-type-specific. They found 2088 and 574 differentially and specifically expressed genes in one cell-type in the MCT and SuHx PAH-model, respectively. For example, IL6ST was upregulated in a subpopulation of arterial endothelial cells from SuHx mice. IL-6 was explicitly upregulated in non-conventional monocytes in SuHx-mice and neutrophils in MCT rats, suggesting model and species-specific differences in the IL-6 signaling. GPR15, which encodes an orphan G-protein-linked receptor, was exclusively upregulated in SuHx-regulatory T cells (Tregs) and is implicated in Treg homing [190]. Distinct profiles were identified in the two rodent models of PAH. Strong downregulation of interferon (IFN) signaling was observed in the MCT model, while slightly upregulated in the SuHx model. Such differences in the gene expression profile further emphasized the molecular complexity of the pathomechanisms in PAH, as well as the dissimilarities and limitations of the commonly used animal models of PAH. This study also demonstrated the power of scRNA-Seq-based approaches in the identification of differentially expressed genes and pathways in a cell-type-specific manner [190].

### 9.2. Artificial Intelligence (AI)

One proposed application of such technologies is to use routinely collected patient data to predict patients at high risk for disease and improve patient outcomes [191]. To mine into large data sets, a screening algorithm is the most crucial aspect of using big data with AI. The algorithms are usually developed through numerous iterative steps by multi-disciplinary teams, including clinical and artificial intelligence experts. Recently, a study from Sheffield Pulmonary Hypertension index (SPHInX) demonstrated that patients with IPAH showed higher levels of healthcare utilization in a three-year period before diagnosis, with around 25 hospital visits. Using their AI screening tool, the authors were able to have a specificity of 99.99% and a sensitivity of 14.10%. The model also predicted patients being at high risk of iPAH 1151 times more often in patients with IPAH than patients who did not [192]. This study provided critical insights into how big data and machine learning using AI can be utilized to predict and identify patients with a high risk of disease before they are diagnosed. This is an untapped area in healthcare with immense potential for improving patient outcomes and healthcare.

## 10. Conclusions

In the past decades, extensive research effort has contributed to tremendously improve our understanding of PAH since the initial BMPR2 mutation was identified. The development of new multi-omics approaches and NGS technologies have propelled our progress even further and led to the implementation of precision medicine. By generating individualized omics signature, personalized medicines provide valuable information to guide health care decisions toward the most appropriate and effective targeted therapies. The characterization of mutational signatures has been of great interest in the field of PAH. Mutation prevalence and profiling have provided a more comprehensive understanding of the multifactorial nature of PAH and opened new avenues for the design of novel therapeutic approaches. The complex nature of PAH, genetic variation in association with environmental pressures requires an inherent understanding of molecular events. Along with the recent development of new research techniques, new diagnostic tools, and precision medicine that provides individual characteristics of each PAH patient, numerous emerging therapies have shown promising results in preventing and reversing PAH in preclinical models. Ongoing clinical trials are defining the therapeutic potential of epigenetic and genomic medicines with the hope that, shortly, cutting-edge medicines and therapies will be available to patients affected by this fatal disease.

## Figures and Tables

**Table 1:** This table entails the genetic variants in PAH, the respective affected location, domain, pathway, and phenotype.

Gene	Location	Type of Mutation		Domain	Pathway	Phenotype	Animal Model	Ref
**BMPR2**	Exon 1	Missense—Nonsense	Loss of Function	Extracellular Domain	SMAD1/5/8 TGF-β	↑ RV remodeling ↑ Vascular remodeling ↑ mPAP ↑ PVR	RatMice	[38,126,127]
	Exon 2	Nonsense Deletion Missense						
	Exon 3	Missense—Nonsense Frameshift Insertion Deletion						
	Exon 4	Deletion						
	Exon 5	Missense—Nonsense		Transmembrane Domain				
	Exon 6	Frameshift Deletion Frameshift Insertion Nonsense—Missense		Kinase Domain				
	Exon 7	Nonsense—Missense						
	Exon 8	Missense—Nonsense Frameshift Deletion Deletion						
	Exon 9	Missense—Nonsense Frameshift Deletion						
	Exon 10	Nonsense Frameshift Deletion						
	Exon 11	Missense						
	Exon 12	Nonsense Frameshift Deletion		Cytoplasmic Domain				
	Exon 13	Missense						
**TBX4**	Exon 3 Exon 8	Missense—Deletion	Loss of Function	DNA Binding Domain	FGF10 BMP4	↑ Vascular remodeling	MiceChicken	[113,114]
**ALK1**	Exon 2 Exon 5	Missense	Loss of Function	Extracellular Domain	BMP9 TGFB1 BMPR2	↑ RV hypertrophy ↑ Vascular remodeling ↑ mPAP	Mice	[27,72,128,129]
	Exon 7 Exon 8 Exon 9 Exon 10			Kinase Domain				
**ENG**	Exon 5 Exon 6 Exon 10 Exon 11 Exon 12	Missense	Loss of Function	Extracellular Domain	SMAD1/5/8 BMP9/10 BMPR2	↑ Vascular remodeling	Mice	[57,59,128,130]
**EIF2AK4**	Exon 38	Nonsense Deletion Frameshift Deletion	Loss of Function	Kinase Domain	TRIB3 EIF2S1 BMPR2 SMAD1/5/8	↑ Inflammation ↓ Autophagy ↑ ROS	Mice	[88,89,90]
**KCNK3**	Exon 2	Missense	Loss of Function	Pore Domain Transmembrane Domain	KCNK9 VGCC STAT3	↑ Vascular remodeling ↑ Vasoconstriction	Mice	[103,104,105,106,107,131,132]
**CAV1**	Exon 1 Exon 2 Exon 3	Frameshift deletion	Loss of Function	C-Terminal Domain	ENOS PKG-1	↑ Vascular remodeling ↑ PASMC proliferation and migration ↑ Vasoconstriction	Mice	[68,91,98,100,101,102]
**TET2**	Exon3	Missense	Loss of Function	Catalytic Domain	IL-1B	↑ Inflammation	Mice	[118]
**GDF2**	Exon 1	Missense Nonsense Frameshift Deletion	Loss of Function	Prodomain	IL-6 BMP10 BMPR2	↑ Inflammation ↓ Autophagy	Humans	[54]
